# Effect of Shoulder Flexion Range of Motion and Trunk Muscle Activity on Lumbar Lordosis in the Streamlined Posture in Healthy Young Men

**DOI:** 10.7759/cureus.72958

**Published:** 2024-11-04

**Authors:** Masahiro Sawada, Yusuke Kubo, Tetsuya Kageyama, Kohtaro Yamashita, Shuhei Sugiyama, Rie Takachu, Takeshi Sugiura, Kaori Kobori, Makoto Kobori, Makoto Nejishima

**Affiliations:** 1 Department of Rehabilitation Medicine, Kobori Orthopedic Clinic, Hamamatsu, JPN; 2 Department of Physical Therapy, Chuo Medical Health Vocational College, Shizuoka, JPN; 3 Department of Orthopedics, Kobori Orthopedic Clinic, Hamamatsu, JPN; 4 Department of Rehabilitation Sciences, Seirei Christopher University, Hamamatsu, JPN

**Keywords:** low back pain, lumbar lordosis angle, shoulder flexion restriction, streamlined posture, swimming

## Abstract

Objective

This study aimed to compare the lumbar spine, pelvic alignment, and trunk muscle activity in healthy participants with and without shoulder flexion restriction in a streamlined posture.

Methods

The lumbar lordosis angle, pelvic anteversion angle, and trunk muscle activity were measured in 31 young, healthy men in the resting standing position. The streamlined (SL) posture and correlation analysis were performed. Multiple regression analysis was performed to analyze variables that affect the lumbar lordosis angle in the SL posture.

Results

Correlation analysis revealed that the lumbar lordosis angle in the SL posture had a significant negative correlation with the muscle activity of the internal oblique/transversus abdominis muscle (IO/TrA) during the SL posture (r=-0.37, p<0.045). Multiple regression analysis revealed that lumbar lordosis angle in the SL posture was associated with lumbar lordosis angle in the upright position (β=0.46), IO/TrA activity (β=-0.37), shoulder joint flexion range of motion (β=-0.37), and longissimus dorsi muscle activity during the SL posture (β=-0.28), with a Durbin-Watson statistic adjusted coefficient of determination R^2^ of 0.67 (p<0.001).

Conclusion

The lumbar lordosis angle in the SL posture was likely to be high when the range of motion of shoulder joint flexion was small, the activity of the longissimus dorsi muscle in the SL posture was high, and the activity of the IO/TrA was low. This suggests that limited shoulder flexion causes excessive lumbar lordosis, which may be a mechanism of low back pain in swimmers.

## Introduction

Swimming, a sport characterized by frequent trunk flexion, extension, and rotation, is associated with a high incidence of low back pain. A survey conducted among swimmers revealed that lower back pain was the most prevalent injury, with 19% of top junior swimmers and 17% of swimmers designated by the Japan Swimming Federation reporting a history of lower back pain within the past year [1,2]. Low back pain is a chronic musculoskeletal disorder that can affect swimmers of all ages. As such, it is crucial to implement measures aimed at preventing and mitigating the occurrence of low back pain in order to maintain performance and sustain competitive swimming.

Lumbar lordosis has been identified as a potential cause of low back pain among swimmers, specifically in the streamlined (SL) posture, where the upper limbs are elevated vertically. The SL posture is a fundamental position in swimming, particularly during the start and turn phases, and is characterized by the swimmer's body being fully extended to minimize drag in the water. Previous research has found that lumbar lordosis in the SL posture increases the load on the posterior elements of the lumbar spine, which is considered a risk factor for developing low back pain [3,4]. The diverse nature of swimming events, in which swimmers do not exclusively practice specific techniques, poses challenges to completely understanding the mechanism of low back pain during each event [5]. The SL posture, a fundamental posture shared across all swimming techniques and frequently executed during the start and turn phases, offers a possible area of investigation. Therefore, clarifying the underlying cause of lumbar lordosis in the SL posture should facilitate a better understanding of the mechanism of low back pain in swimmers.

It has been proposed that multiple factors, such as limited shoulder flexion, increased anterior pelvic tilt angle, and decreased activity of deep trunk muscles, may contribute to lumbar lordosis in the SL posture. Research investigating the relationship between shoulder and thoracic mobility and lumbar lordosis in the SL posture found a negative correlation between shoulder mobility and lumbar lordosis angle; individuals with less shoulder mobility exhibited greater lumbar lordosis [6]. Regarding the lumbar spine and pelvic alignment during the SL posture, the lumbar lordosis angle and pelvic anterior tilt angle increased compared with the upright posture [7]. It has been reported that the lumbar lordosis angle increases when the transversus abdominis muscle, a deep trunk muscle, is less active, both in standing and in the SL posture, with these effects observed during measurements conducted while in water [8]. The transversus abdominis muscle is involved in tilting the pelvis backward [9,10], leading to a reduction in the lumbar lordosis angle [11], which helps to regulate lumbar alignment in the SL posture [8]. Therefore, lumbar lordosis in the SL posture may result from limited shoulder flexion, increased anterior pelvic tilt, and decreased deep trunk muscle activity.

Although several factors have been proposed as contributing to increased lumbar lordosis in the SL posture [12], no previous studies have simultaneously examined the effect of shoulder joint range of motion (ROM), pelvic alignment, and trunk muscle activity on lumbar lordosis in this posture. This study aims to investigate how these factors influence lumbar lordosis in young, healthy men, providing a clearer understanding of the mechanisms behind lumbar lordosis in the SL posture.

## Materials and methods

Subjects

The participants were 36 male university students (age range: 18-22 years) enrolled at a medical college. We selected healthy individuals without any history of injuries or functional impairments, such as joint ROM limitations or muscle weakness. Importantly, none of the participants were regular swimmers or engaged in aquatic training. This was intentional because swimmers often experience specific loads on their shoulders and other joints due to aquatic training. The repetitive movements in a weightless environment can lead to increased joint mobility in the shoulders and other anatomical regions [13,14]. By selecting non-swimmers, we aimed to eliminate the influence of physical characteristics and to clarify the effect of limited shoulder joint flexion on postural alignment during the execution of the SL posture. Participants with a body mass index (BMI) outside the standard range [15] of 18.5 ≤ BMI < 25.0 kg/m^2^ or with a history of orthopedic diseases of the shoulder joint, knee joint, hip joint, or spine were excluded to minimize differences in body size among healthy participants. All participants underwent physical screening prior to the study to ensure there were no joint dysfunctions or muscle weaknesses that might have influenced the results. The screening tests for physical characteristics included joint ROM measurements and manual muscle testing (MMT).

**Figure 1:**
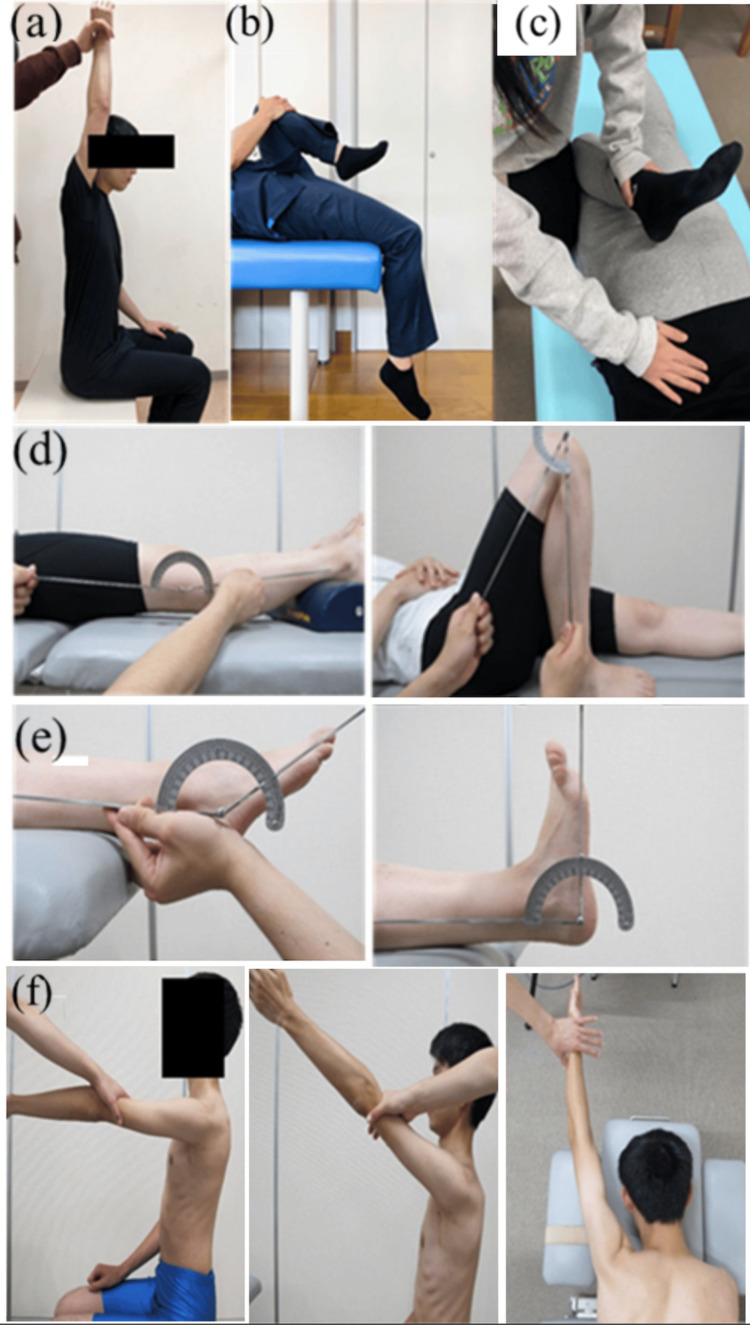
The screening tests for physical characteristics (a) Shoulder flexion, (b) Thomas test, (c) heel-buttock distance (HBD), (d) knee joint extension and flexion, (e) ankle joint in plantar flexion and dorsiflexion, (f) manual muscle testing (MMT): anterior deltoid, serratus anterior, and lower trapezius muscles.

The ROM of the shoulder joint was measured in flexion, the hip joint was evaluated using the Thomas test, the heel-buttock distance (HBD) was measured, and the knee joint was measured in both extension and flexion, while the ankle joint was assessed in plantar flexion and dorsiflexion. The Thomas test was considered negative if there was no compensation by hip flexion on the side contralateral to the test side and positive if there was compensation by hip flexion on the test side. The HBD test was considered negative if the calcaneus and hip on the test side could be touched, negative if there was no hip flexion or hip elevation, and positive if the calcaneus and hip could not be touched or the hip was elevated. A difference of less than 5° between the right and left knee joint extension and flexion was considered negative, while a difference of 5° or greater was considered positive. The MMT was performed on the anterior deltoid, serratus anterior, and lower trapezius muscles. The criteria for muscle strength testing were evaluated according to the new manual muscle strength testing method [16], with negative results for an MMT grade of 5 or greater and positive results for an MMT grade of 5 or lesser (Figure 1).

Following an experimental protocol and informed consent form approved by the Seirei Christopher University Ethics Committee (approval no.: 20030), the participants were thoroughly informed of the objectives and methodology of the research, and informed consent was obtained before beginning the measurements.

Measurement

**Figure 2:**
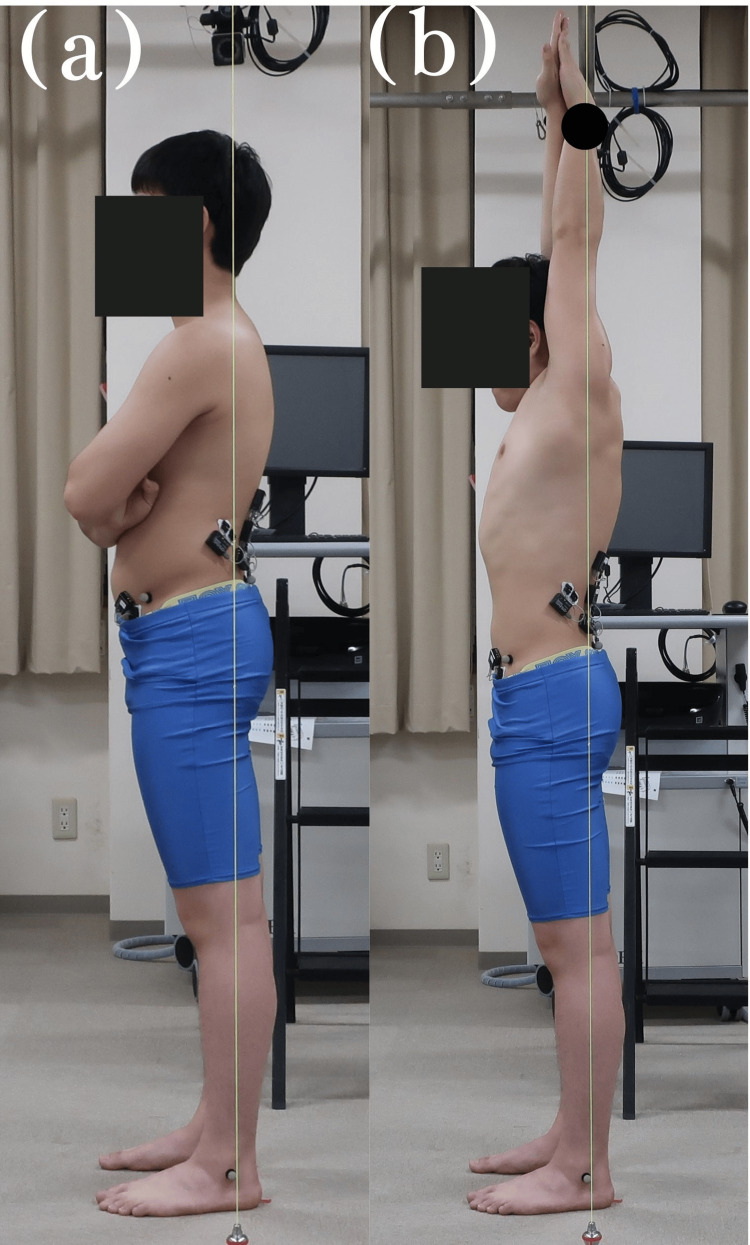
Resting standing position and the streamlined posture on land (a) The resting standing position; (b) the streamlined posture with the ulnar stem cell process overlapping the perpendicular line to the floor; the black circle indicates the ulnar stem cell.

Two experimental conditions were employed for the measurements: (a) the resting standing position and (b) the SL posture on land (Figure 2). In the SL posture, the participants' elbows were extended, and both shoulder joints were flexed from their resting standing position. To standardize the flexion angles of both shoulder joints, a reflex marker was affixed to the participant's left ulnar eminence, and both shoulder joints were flexed to the same position as the downward swing suspended from the ceiling post to the floor. The SL posture was practiced several times to confirm that the participants could maintain the posture with their bodies held in a straight line. The SL posture was determined by a physical therapist with a minimum of five years of clinical experience at an orthopedic surgery clinic. The measurement procedure started with the resting standing position, where the lumbar lordosis angle and pelvic tilt angle were measured. Then, the participants assumed the SL posture, and the trunk muscle activity, lumbar lordosis angle, and pelvic tilt angle were measured. Measurements of the lumbar lordosis angles in the resting standing position and SL posture were performed using two inclinometers (A-300, Shinwa Rules Co., Ltd. Niigata, Japan). The lumbar lordosis angle was measured using an inclinometer, and it was reported to have moderate validity in a previous study, with an intra-examiner reliability of 0.90 and an inter-examiner reliability of 0.85 [17,18]. An inclinometer was adopted because it avoids the use of radiography, thereby eliminating concerns regarding radiation exposure, and is easy and efficient to use in clinical settings.

**Figure 3:**
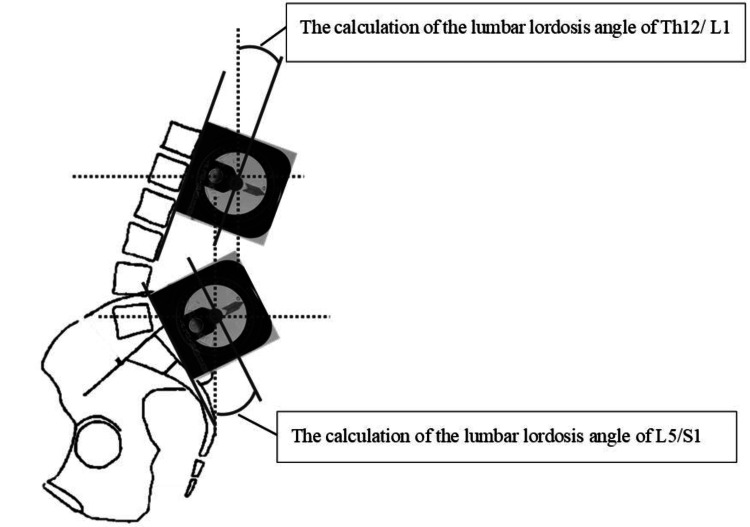
Method of calculating the lumbar lordosis angle The calculation of the lumbar lordosis angle in the streamlined posture (SL) was derived using the following formula: lumbar lordosis angle in SL posture (°) = lumbar lordosis angle of T12/L1 + lumbar lordosis angle of L5/S1. Image created by the authors.

The inclinometer was manually positioned along the spinal column between the 12th thoracic vertebra and the first lumbar vertebra (T12/L1) and between the fifth lumbar vertebra and the sacral vertebra (L5/S1), as illustrated in Figure 3. The locations of the first and fifth lumbar spinous processes were identified by a physical therapist with at least five years of clinical experience at an orthopedic clinic. To accurately measure the angle of anterior pelvic tilt in both the resting standing position and SL posture, digital images were captured in the sagittal plane. Prior to imaging, reflective markers were strategically placed on the participant's left anterior superior iliac spine (ASIS) and left posterior superior iliac spine (PSIS). To control and eliminate camera tilt, the digital camera (PowerShot G12, Canon Marketing Japan Inc., Tokyo, Japan) was mounted on a tripod and leveled using an inclinometer to ensure both the horizontal and vertical axes were set to 0°. The camera was positioned perpendicular to the participant's sagittal plane at a fixed distance, ensuring consistent framing. Additionally, a plumb line suspended from the ceiling was included in the field of view as a vertical reference to confirm proper alignment in the images. The distance between the camera and the participant, as well as the camera height, were adjusted so that the participant was centered in the angle of view during both the resting standing and SL postures. To prevent any camera tilting, an inclinometer was used to set the front-back tilt and lateral tilts to 0°. Six digital images were obtained: three each from the resting standing and SL positions.

Wireless electromyography (Telemyo EM2015R, Sakai Medical Co., Ltd. Tokyo, Japan) was used to measure trunk muscle activity in the SL posture. The five muscles measured were the internal oblique/transversus abdominis (IO/TrA), longissimus thoracis (LT), longissimus lumborum (LL), iliocostalis lumborum (IL), and multifidus (MF) on the left side. For the IO/TrA, the electrodes were placed parallel to the muscle fibers, 20 mm medial to and below the superior anterior iliac spine [19,20]. The electrode for the LT was placed 30 mm lateral to the T12 spinous process [21]. This electrode was placed parallel to the LT muscle fibers. The electrode for the LL muscle was placed 30 mm lateral to the L1 spinous process. The electrode for the MF muscle was placed 20 mm lateral to the L5 spinous process and at the intersection of the line connecting the L1 spinous process and the superior posterior iliac spine. The distance between the electrode centers was 20 mm. The activity of each muscle was recorded three times during the five-second holding period following the completion of the SL posture.

Analysis

**Figure 4:**
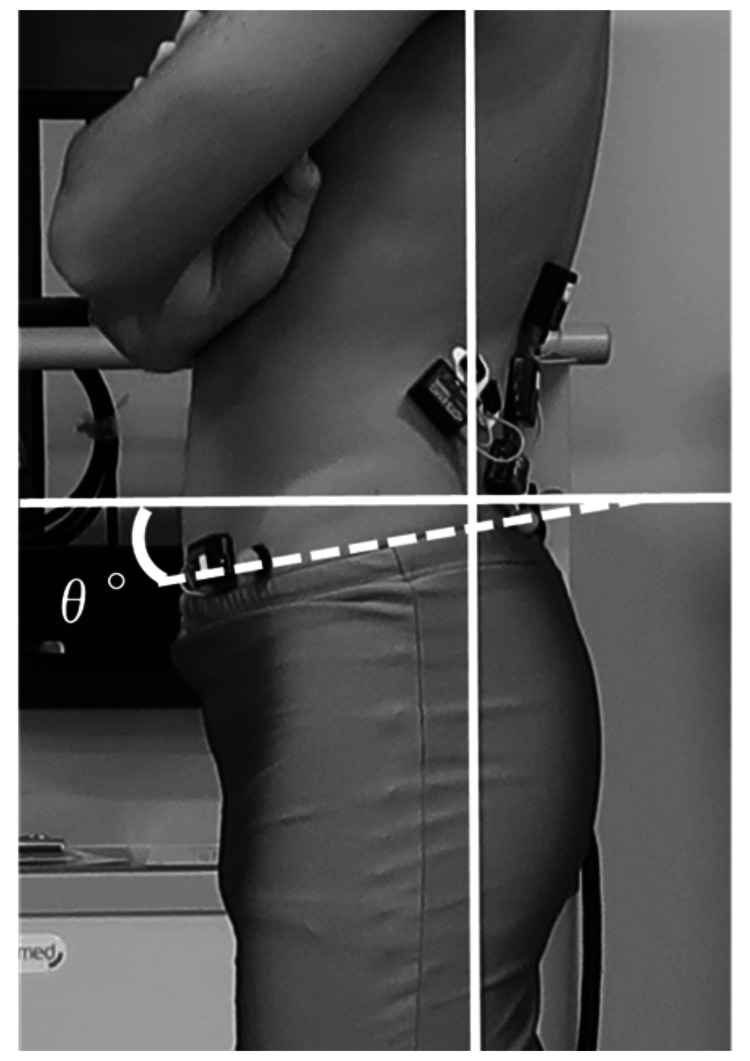
Calculation of pelvic anteversion angle The angle (θ°) representing the degree of anterior pelvic tilt in the streamlined posture was established as the angle formed by the horizontal line connecting the left upper anterior iliac spine and the left upper posterior iliac spine (white dotted line).

The lumbar lordosis angle was calculated using the inclinometer angle for each condition. The upper and lower lumbar lordosis angles were added to obtain the lumbar lordosis angle (Figure 3). An image analysis software (ImageJ, version 1.43u; National Institute of Health, Bethesda, MD, USA) was used to calculate the pelvic anteversion angle under each condition. The angle (θ°) formed by the horizontal line connecting the left ASIS and left PSIS in the sagittal plane was calculated as the pelvic anteversion angle in the SL posture (Figure 4). The average of the three pelvic anteversion angles obtained for each condition was used as the representative value. The integral values of each muscle in the SL position were calculated from the obtained electromyograms. The central three seconds of the five-second muscle activity were included in the analysis. The integrated value for each muscle was the average of three representative values. The maximal voluntary contraction (MVC) of each muscle was performed before the measurement of trunk muscle activity to normalize the integrated values for each condition. For the LT, LL, IL, and MF muscles, the participants were placed in the supine position with their hands clasped behind their heads to maximally extend the spinal column. The participant was instructed to lift his entire rib cage off the bed, and resistance was applied to his upper back (around the scapula) in the direction of spinal flexion. Another evaluator held the lower leg to prevent it from lifting when a force was exerted. For the IO/TrA muscles, the participant was placed in a supine position, and a full-force draw-in was performed. The participants were asked to perform a draw-in that fully activated the IO/TrA muscles while checking the electromyogram monitor, and the measurements were obtained after sufficient practice [22]. Three maximal isometric contractions of five seconds each were performed, and the average was taken as the representative value. The %MVC was calculated by dividing the integral value obtained for each condition by the obtained MVC.

Statistical analysis

The Shapiro-Wilk test was used to confirm the normality of the data before statistical analysis. Correlation analysis was then used to analyze lumbar lordosis angle, shoulder flexion ROM, pelvic anteversion angle, and trunk muscle activity in the SL posture. Pearson's correlation analysis was performed when normality was found, and Spearman's correlation analysis was performed when normality was not found. Subsequently, a linear multiple regression analysis was conducted to identify factors affecting the lumbar lordosis angle in the SL posture. The dependent variable was the lumbar lordosis angle in the SL posture, and the independent variables were shoulder flexion ROM, pelvic anteversion angle in the SL posture, and trunk muscle activity in the SL posture. In this study, exploratory research was needed to clarify the relationship between the cause of back pain in swimmers and lumbar lordosis in the SL posture. When a variable was not used as a variable in the correlation analysis, the p-value was set at 0.1, and the variable was forced entry method. IBM SPSS Statistics for Windows, Version 24 (Released 2016; IBM Corp., Armonk, New York) was used for statistical analysis, and the significance level was set at a 5% risk rate.

## Results

The results of the physical characteristics screening test revealed that 31 of the 36 participants met the established criteria (Table 1), and five were excluded (two participants had current low back pain, one had a history of multiple orthopedic conditions and a BMI exceeding the standard range, and two had a BMI exceeding the standard range). Normality was observed in the lumbar lordosis angle in the SL posture, shoulder joint flexion ROM, and pelvic anteversion angle in the SL posture. No normality was observed in the activity of the trunk muscles during the SL posture.

**Table 1:** The results of the screening test for physical characteristics IQR: interquartile range; BMI: body mass index; HBD: hip buttock distance; MMT: manual muscle testing; ROM: range of motion

Demographic and Clinical Characteristics	Median (IQR)/n (%)
Age, median (IQR)	21.3 (20.0-22.0)
Height (m), median (IQR)	1.71 (1.64-1.75)
Weight (kg), median (IQR)	62.6 (59.6-66.9)
BMI (kg/m^2^), median (IQR)	22.2 (20.6-23.7)
Shoulder flexion angle (°), median (IQR)	169.0 (165.8-171.3)
MMT	-
Anterior deltoid, positive, n (%)	0 (0.0)
Serratus anterior, positive, n (%)	0 (0.0)
Lower trapezius muscles, positive, n (%)	11 (35.5)
Hip flexibility	-
Modified Thomas test, positive (%)	6 (19.4)
HBD, positive (%)	20 (58.1)
ROM	-
Knee Joint, positive (%)	1 (3.2)
Hip Joint, positive (%)	0 (0.0)

Correlation analysis revealed that the lumbar lordosis angle in the SL posture had a significant negative correlation with the muscle activity of the IO/TrA during the SL posture (Table 2). The pelvic anteversion angle in the SL posture had a significant positive correlation with the activities of the LT, LL, IL, and MF in the SL posture (Table 3). Multiple regression analysis showed that the following items were associated with lumbar lordosis angle in the SL posture: standing lumbar lordosis angle (β=0.46), shoulder flexion ROM (β=-0.65), LT activity in the SL posture (β=0.40), and IO/TrA activity (β=-0.12), with an adjusted coefficient of determination R2 of 0.61 (p<0.001) (Table 4). The regression equation was: lumbar lordosis angle in the SL posture = 116.292 + 0.455 × standing lumbar lordosis angle - 0.37 × IO/TrA activity - 0.37 × shoulder joint flexion ROM + 0.28 × LT activity in the SL posture. The Dubrin-Waston statistic was 1.19, indicating that the residual autocorrelation of the model was minimal. Additionally, the variance inflation factor (VIF) for all independent variables was below 5, confirming that there was no significant multicollinearity among the predictor variables, ensuring the reliability of the regression model's coefficients.

**Table 2:** Correlation between the streamlined posture lumbar lordosis angle and various physical activity measures *indicates levels of statistical significance, p < 0.05 ROM: range of motion; IO/TrA: internal oblique/transversus abdominis; LT: longissimus thoracis; LL: longissimus lumborum; IL: iliocostalis lumborum; MF: multifidus muscles; MVC: maximal voluntary contraction

	Shoulder ROM	Muscle Activity of IO/TrA (%MVC)	Muscle Activity of LT (%MVC)	Muscle Activity of LL (%MVC)	Muscle Activity of IL (%MVC)	Muscle Activity of MF (% MVC)	Pelvic Tilt Angle
r	-0.35	-0.37*	0.35	0.17	-0.23	0.07	-0.10
p-value	0.052	0.045	0.051	0.36	0.21	0.70	0.60

**Table 3:** Correlation between streamlined posture pelvic tilt angle and various physical activity measures *indicates levels of statistical significance, p < 0.05 ROM: range of motion; IO/TrA: internal oblique/transversus abdominis; LT: longissimus thoracis; LL: longissimus lumborum; IL: iliocostalis lumborum; MF: multifidus muscles; MVC: maximal voluntary contraction

	Shoulder ROM	Muscle Activity of IO/TrA (%MVC)	Muscle Activity of LT (%MVC)	Muscle Activity of LL (%MVC)	Muscle Activity of IL (%MVC)	Muscle Activity of MF (% MVC)
r	0.38*	-0.02	0.41	0.53*	0.46*	0.55*
p-value	0.04	0.93	0.02^*^	0.002	0.01	0.001

**Table 4:** Results of multiple regression analysis for factors affecting lumbar lordosis angle in the streamlined posture This table shows the results of a multiple regression analysis predicting factors that increase the lumbar lordosis angle in the streamlined posture. Independent variables include lumbar lordosis angle in the standing posture (°), muscle activity of the IO/TrA (%MVC), shoulder joint flexion ROM (°), and muscle activity of the LT (%MVC). Standardized beta coefficients, p-values, and VIF are reported for each independent variable. Each independent variable had a p-value of less than 0.05, indicating a significant difference. ROM: range of motion; SL: streamlined posture; LT: longissimus thoracis; IO/TrA: internal oblique/transversus abdominis; VIF: variance inflation factor

Independent Variable	Standardized Coefficient (β)	P-value
Lumbar lordosis angle (standing)	0.46	<0.001
Shoulder flexion ROM	-0.65	<0.001
Muscle activity of LT in SL posture	0.40	0.02
Muscle activity of IO/TrA in SL posture	-0.12	0.03
Adjusted R^2^	0.61	-
Durbin-Watson statistic	1.19	-

## Discussion

This study is clinically useful in that the lumbar lordosis angle in the SL posture was examined simultaneously with shoulder joint flexion ROM, pelvic anteversion angle, and trunk muscle activity in young, healthy male participants, and the relationship between each variable was clarified. The results of this study showed that the lumbar lordosis angle in the SL posture increased when the ROM of the shoulder joint flexion was high, the activity of the IO/TrA in the SL posture was small, and the activity of the LT was large, as hypothesized.

A significant negative correlation was observed between the lumbar lordosis angle in the SL posture and shoulder joint flexion ROM. Participants with limited shoulder flexion ROM exhibited increased lumbar lordosis angles in the SL posture. This finding suggests that when shoulder flexion ROM is restricted, the body compensates by increasing lumbar lordosis to achieve the necessary shoulder positioning during the SL posture. These results are consistent with previous studies. Kataura et al. [6] and Suzuki et al. [12] reported that swimmers with greater shoulder flexion ROM have smaller lumbar lordosis angles and experience less alteration when assuming the SL posture. Generally, shoulder flexion is facilitated by coordinated movements involving scapular upward rotation, thoracic spine extension, lumbar lordosis, and anterior pelvic tilt [23,24]. When shoulder joint flexion ROM is limited, adequate scapular motion and thoracic extension cannot occur, necessitating an increase in lumbar lordosis angle as a compensatory mechanism. Therefore, in the SL posture, individuals with restricted shoulder flexion ROM tend to compensate by increasing their lumbar lordosis angle.

We found that in the SL posture, higher muscle activity of the LT and lower muscle activity of the IO/TrA were significantly associated with an increased lumbar lordosis angle. It has been reported that trunk motion during shoulder flexion increased trunk extension and erector spinae muscle activity [25,26] and increased the lumbar lordosis angle [11,27]. The MF functions primarily in thoracolumbar extension and postural maintenance, while the IL attaches from the pelvis to the ribs and plays an auxiliary role [28]. In contrast, the muscular activity of the IO/TrA tilts the pelvis backward and controls the increase in the lumbar lordosis angle [9,10]. It is presumed that in the SL posture, the IO/TrA muscles were unable to control the excessive increase in lumbar lordosis angle. This excessive lumbar lordosis may be due to shoulder joint flexion restriction, which was compensated for by the increased activity of the LT muscle and an increase in lumbar lordosis angle.

In previous studies, it has been considered that low back pain in swimmers is caused by the SL posture with lumbar lordosis of the lumbar spine due to limited shoulder joint flexion ROM, increased pelvic forward tilt angle, and decreased muscle activity of the IO/TrA. The results of this study indicate that the lumbar lordosis angle in the SL posture is likely to be increased when the shoulder joint flexion angle is small, the activity of the LT in the SL posture is large, and the activity of the IO/TrA is small. This result supports the mechanism of low back pain in swimmers, which has been discussed in previous studies. In the case of limited shoulder flexion, compensatory motion may have occurred depending on excessive lordosis of the lumbar spine. If swimmers continue to practice and compete with this compensatory motion, excessive mechanical stress is applied to the posterior lumbar tissues, which is likely to lead to low back pain with organic changes. Interventions to improve the shoulder joint flexion ROM and the coordination of the abdominal and back muscle groups in the SL posture are considered important. This will reduce the incidence of low back pain in swimmers and help them continue to compete in the long term. Future studies on swimmers with low back pain and the effects of changes in trunk muscle activity and fatigue will help to elucidate the mechanism of low back pain occurrence.

Based on these findings, specific interventions can be recommended for the training and rehabilitation of swimmers to prevent low back pain. Improving shoulder joint flexion ROM through targeted flexibility exercises and stretching programs can reduce the need for compensatory lumbar lordosis during the SL posture. Additionally, strengthening the IO/TrA muscles can enhance pelvic stability and control of lumbar lordosis angle, minimizing excessive lumbar curvature. Incorporating exercises that promote coordinated activation of the abdominal and back muscle groups may help swimmers maintain optimal spinal alignment during swimming activities. These interventions can be integrated into swimmers' regular training routines to reduce the incidence of low back pain, enhance performance, and contribute to the longevity of their athletic careers.

This study has several potential limitations: first, the absence of measurements of the scapulothoracic joint. Because we did not analyze scapulothoracic joint or thoracic spine motion, the effect of shoulder flexion limitation on SL posture was not fully investigated. Second, all measurements in this study were performed on land, and it is important to recognize that the effect of gravity on the SL posture may differ significantly from that in water. Moreover, the buoyancy in water may have a greater impact on muscle tone than on land. Third, the participants in this study were young, healthy men with no prior swimming experience. A previous study suggested that there may be a difference in lumbar lordosis during shoulder flexion between experienced and inexperienced swimmers [23], with experienced swimmers possibly having a greater ROM in the shoulder and other joints than normal [13]. This difference is an important factor to consider when determining training programs and back pain prevention for experienced competitive swimmers. Therefore, it is important to acknowledge that the compensatory motion of swimmers with limited shoulder flexion may differ from that of non-swimmers in the SL posture. Fourth, the lumbar lordosis angle and pelvic inclination angle were not measured using X-ray imaging, which is considered the gold standard for spinal alignment assessment. Instead, we used inclinometers and digital image analysis to avoid radiation exposure and for practical applicability in clinical settings. While these methods have been reported to have moderate to high reliability [17,18], they may not provide the same level of accuracy as radiographic measurements. This limitation could affect the precision of our measurements and the validity of the findings. Future studies should consider using radiographic imaging to obtain more accurate assessments of spinal alignment.

The lumbar lordosis angle in the SL posture in young, healthy men is likely to be larger when the shoulder joint flexion angle is small, the longissimus dorsi muscle activity in the SL posture is large, and the IO/TrA activity is small. Restriction of shoulder flexion ROM increases the lumbar lordosis angle in the SL posture, and excessive lumbar lordosis can cause low back pain. In the future, it would be of more clinical significance if we could analyze the SL posture in patients with shoulder joint disorders and low back pain and verify the relationship between shoulder joint flexion limitation and spinal and pelvic alignment in the SL posture.

## Conclusions

In this study, we investigated the effects of shoulder flexion ROM, pelvic anteversion angle, and trunk muscle activity on lumbar lordosis angle in the SL posture in young, healthy male participants without functional impairment such as joint ROM limitation or muscle weakness except in the shoulder joint. The lumbar lordosis angle in the SL posture is likely to increase when the shoulder flexion angle is small, the activity of the LT is high, and the activity of the IO/TrA is low. This suggests that limited shoulder flexion causes excessive lumbar lordosis, which may be a mechanism for the occurrence of low back pain in swimmers.
